# Investigating the Respiratory and Energy Metabolism Mechanisms behind ε-Poly-L-lysine Chitosan Coating’s Improved Preservation Effectiveness on *Tremella fuciformis*

**DOI:** 10.3390/foods13050707

**Published:** 2024-02-26

**Authors:** Junzheng Sun, Yingying Wei, Longxiang Li, Baosha Tang, Yanrong Yang, Zheng Xiao, Junchen Chen, Pufu Lai

**Affiliations:** 1 Institute of Food Science and Technology, Fujian Academy of Agricultural Sciences, Fuzhou 350003, China; sunjzll@163.com (J.S.); wyy787459024@163.com (Y.W.); 13616901013@163.com (L.L.); tbsty@126.com (B.T.); 18960973035@163.com (Y.Y.); ethyxwat@163.com (Z.X.); junchenccc@163.com (J.C.); 2 National R&D Center for Edible Fungi Processing, Fuzhou 350003, China; 3 Key Laboratory of Subtropical Characteristic Fruits, Vegetables and Edible Fungi Processing (Co-Construction by Ministry and Province), Ministry of Agriculture and Rural Affairs, Fuzhou 350003, China; 4 Institute of Postharvest Technology of Agricultural Products, College of Food Science, Fujian Agriculture and Forestry University, Fuzhou 350002, China; 5 College of Life Science, Fujian Agriculture and Forestry University, Fuzhou 350002, China

**Keywords:** ε-poly-L-lysine, chitosan, *Tremella fuciformis*, respiratory metabolism, energy metabolism

## Abstract

Freshly harvested *Tremella fuciformis* contains high water content with an unprotected outer surface and exhibits high respiration rates, which renders it prone to moisture and nutrient loss, leading to decay during storage. Our research utilized ε-poly-L-lysine (ε-PL) and chitosan as a composite coating preservative on fresh *T. fuciformis*. The findings revealed that the ε-PL + chitosan composite coating preservative effectively delayed the development of diseases and reduced weight loss during storage compared to the control group. Furthermore, this treatment significantly decreased the respiration rate of *T. fuciformis* and the activity of respiratory metabolism-related enzymes, such as alternative oxidase (AOX), cytochrome c oxidase (CCO), succinic dehydrogenase (SDH), 6-phosphogluconate dehydrogenase, and glucose-6-phosphate dehydrogenase (6-PGDH and G-6-PDH). Additionally, the composite coating preservative also delayed the depletion of ATP and ADP and maintained higher levels of the energy charge while preserving low levels of AMP. It also sustained heightened activities of Mg^2+^-ATPase, Ca^2+^-ATPase, and H^+^-ATPase enzymes. These results demonstrate that utilizing the ε-PL + chitosan composite coating preservative can serve as a sufficiently safe and efficient method for prolonging the shelf life of post-harvest fresh *T. fuciformis*.

## 1. Introduction

*Tremella fuciformis* (also known as snow fungus) is globally acknowledged as a highly nutritious edible fungus, often lauded as the “crown of fungi” [1,2]. Rich in gelatinous substances, amino acids, minerals, and other nutrients, *T. fuciformis* possesses medicinal and health benefits, including blood sugar reduction, lipid-lowering, immune modulation, anti-aging effects, and antiviral effects [3]. China, a major producer and consumer of edible fungi, with a diverse variety and a long history of mushroom cultivation, leads the world in terms of both cultivation area and production of *T. fuciformis* [4,5]. Owing to its high water content, lack of protective cuticle, and active respiration during storage, fresh *T. fuciformis* is particularly prone to moisture loss, nutrient decomposition, spoilage, and the production of toxic substances, thus losing its edible and economic value [6]. The storage of fresh *T. fuciformis* presents significant challenges, yet there is a growing consumer demand for fresh produce. Consequently, the investigation and improvement of preservation methods for *T. fuciformis* is essential to extend its shelf life, reduce wastage, and maintain its nutritional value.

In the preservation of fruits, vegetables, and edible fungi, respiratory metabolism is one of the critical factors influencing the effectiveness of preservation. Respiration, a fundamental biochemical process, is involved not only in energy production but also in a series of complex metabolic pathways, including the metabolism of carbohydrates, fats, and proteins [7,8]. During the preservation of *T. fuciformis*, an increase in the respiratory metabolism rate is often associated with quality degradation and reduced lifespan. Therefore, controlling the respiratory intensity of *T. fuciformis* is crucial for maintaining its freshness and nutritional value. Moreover, energy metabolism plays an essential role in preserving the cellular vitality and freshness of *T. fuciformis*. Adenosine triphosphate (ATP), along with main metabolites, such as adenosine diphosphate (ADP) and adenosine monophosphate (AMP), is vital in sustaining cellular physiological functions [9,10]. Studies have shown that the levels of ATP and its metabolites vary under different storage conditions, reflecting the energy status and metabolic activity of shiitake mushroom (*Lentinus edodes*) cells [11]. Hence, a thorough investigation of the energy metabolism changes in *T. fuciformis* during storage is important for understanding and improving its preservation outcomes.

ε-Poly-L-Lysine (ε-PL) is a homopolymeric substance comprising lysine residues. ε-PL is characterized by favorable antibacterial properties, biodegradability, thermal stability, and solubility in water. It is non-toxic and harmless to the human body, displays efficient endotoxin removal capabilities, and can prevent the production of toxins by oral bacteria [12,13]. Beyond its applications in the food sector, ε-PL has been extensively researched and applied in fields such as agriculture, pharmaceuticals, and cosmetics [14]. Chitosan, a high molecular weight cationic polysaccharide, is safe, non-toxic, soluble in various organic acids, and exhibits a degree of antimicrobial, antioxidant, and film-forming adsorption properties [15,16]. When used as a food preservative, it can inhibit the growth of microbes, reducing spoilage due to bacterial contamination, and can also form a film on the surface of foods, minimizing moisture loss and delaying direct oxygen contact—thereby suppressing the respiration of fruits and vegetables and reducing nutrient depletion. Additionally, chitosan has antioxidant properties; it can scavenge free radicals such as superoxide anions and inhibit ethylene production, thereby delaying the aging of fruits and vegetables [17]. Edible fungi treated with chitosan retain their original taste and appearance and possess considerable advantages in post-harvest freshness preservation [18,19,20].

Currently, due to ε-PL’s excellent broad-spectrum antimicrobial properties and chitosan’s favorable adsorptive film-forming characteristics—and considering that both substances are approved food additives by the market—they present promising application prospects in the post-harvest preservation of fruits and vegetables. However, the effects of a composite coating preservative made from ε-PL and chitosan on the preservation of fresh *T. fuciformis*, as well as its relationship with energy and respiratory metabolism during storage, remain unclear. Therefore, this study aims to build upon the understanding of the composite coating preservative’s impact on the storage quality of fresh *T. fuciformis*. It intends to delve into the mechanism by which this preservative enhances the shelf life of the fresh *T. fuciformis* through an analysis of changes in energy and respiratory metabolic pathways.

## 2. Materials and Methods

### 2.1. Materials and Treatments

For this study, high-quality *T. fuciformis* cultivated in Gutian County, Fujian Province, was selected as the experimental material. Harvesting was carried out once the *T. fuciformis* reached marketable maturity (cultivated for 90–110 days). The components ε-PL and chitosan implemented in this research were sourced from Zhejiang Yinuo Biotechnology Co., Ltd. (Lanxi, China). The biochemical reagents utilized throughout the assay procedure were uniformly procured from Shanghai Macklin Biochemical Technology Co., Ltd. (Shanghai, China). Furthermore, all enzyme-linked immunosorbent assay (ELISA) kits selected for analyses were acquired from Shanghai Kehansheng Biotechnology Co., Ltd. (Shanghai, China).

Fresh *T. fuciformis* specimens with full caps and uniform size that were free of diseases, pests, and mechanical damage were chosen. In our previous work, it was discovered that a composite coating preservative composed of 150 mg·L^−1^ ε-PL and 5 g·L^−1^ chitosan provided an optimal concentration for *T. fuciformis* preservation. Consequently, this study employed the preservative at this concentration. First, 5 g of chitosan were dissolved in 1 L of deionized water and sonicated for 20 min until completely dissolved. Subsequently, 150 mg of ε-PL was added to the chitosan solution, which was then sonicated for 30 min and left to stand for degassing. This process yielded 150 mg·L^−1^ ε-PL and 5 g·L^−1^ chitosan composite coating preservative.

The fresh *T. fuciformis* specimens were evenly divided into two groups (each group contained 120 specimens of *T. fuciformis*), a control group and a composite coating preservative treatment group (ε-C). Each *T. fuciformis* was uniformly sprayed 15 times with either deionized water, for the control, or the composite coating preservative, for the treatment group, with each spray delivering a dose of 0.5 mL. After the liquid on the surface of each *T. fuciformis* specimen had naturally absorbed and drained, each was placed in a polyethylene film bag, with one *T. fuciformis* specimen per bag, and then deposited in an environment of 25 °C and 80% relative humidity for 5 days. During the period it was deposited, *T. fuciformis* from both the control and ε-C treated groups was sampled daily to obtain the following measurements; each *T. fuciformis* specimen was measured three times.

### 2.2. Determination of Disease Index

The disease index was calculated as the following formula:

$$Disease\;Index=100\times \frac{\sum \left(number\;of\;T.\;fuciformis\;at\;each\;level\times representative\;value\;of\;each\;level\right)}{total\;number\;of\;T.\;fuciformis\times maximum\;representative\;value}$$

The method for determining the level of disease index is shown in Table 1.

### 2.3. Determination of Weight Loss Rate

Referencing the process introduced by Sun et al. [21], weight loss rate was calculated with the gravimetric method as follows:

$$Weight\;Loss\;(\%)=\frac{m-m_{1}}{m}\times 100\%$$

where *m* is the initial fresh weight of the *T. fuciformis* (in grams), and *m_1_* is the fresh weight of the *T. fuciformis* at the time of sampling (in grams). 

### 2.4. Determination of Respiration Rate

Following the procedure introduced by Cliffe-Byrnes and Beirne [22], the respiration rate of the fresh *T. fuciformis* during storage was measured using a fruit and vegetable respirometer (HM-GX, Shandong Hengmei Technology Electronics Co., Ltd., Weifang, China). Before measuring the respiration rate, the test container was sealed tightly, and the respirometer was initiated to undergo a 10-min gas equilibration stabilization procedure. A single specimen of *T. fuciformis* was placed inside the test container, ensuring a tight seal, before commencing the respirometer’s respiration rate measurement program. The program was conducted over a period of 20 min, following which, the results were exported.

### 2.5. Assay of Enzymes Activities Related to Respiratory Metabolism

The detection of alternative oxidase (AOX) enzyme activity was conducted using an ELISA kit. The assay for cytochrome c oxidase (CCO) enzyme activity was performed in accordance with the method developed by Li et al. [23], utilizing an extraction mixture containing PBS buffer, EDTA, and sucrose solution, followed by a thorough reaction with cytochrome c solution, H_2_O_2_ solution, and dimethyl-para-phenylenediamine dihydrochloride. The absorbance was then measured at a wavelength of 510 nm. The assays for succinic dehydrogenase (SDH) activity in this study was conducted by the methodology described by Zhang et al. [24], utilizing an extraction mixture containing PBS buffer, EDTA, sucrose solution, and resorcinol solution, followed by a thorough reaction with phenazine methyl sulfate solution, 2,6-dichlorophenol solution, and gelatin, and then measured at a wavelength of 600 nm. The 6-phosphogluconate dehydrogenase and glucose-6-phosphate dehydrogenase (6-PGDH and G-6-PDH) activity reflected the method of Li et al. [25], the combination solution of PBS buffer, bovine serum albumin, EDTA, and sucrose was employed for enzyme extraction and subjected to thorough reaction in a solution composed of Tris-HCl, MgCl_2,_ and glucose-6-phosphate. The absorbance values were measured at 340 nm. Each enzyme extract was prepared from 1 g of *T. fuciformis* tissue and the absorbance was recorded at 600 nm and 340 nm wavelengths for the respective enzymes. The enzyme activities were evinced by U·mg^−1^ protein.

### 2.6. Determination of Energy Level

The quantification of ATP, ADP, AMP, and energy charge levels was carried out following the methods described by Xue et al. [26]. A total 5 g of *T. fuciformis* tissue was taken and mixed with 25 mL of 0.6 mol·L^−1^ perchlorate acid solution. Following centrifugation, 10 mL of the supernatant was collected. The pH was adjusted to a range from 6.5 to 6.8 using 1 mol·L^−1^ KOH solution; the mixture was then subjected to an ice bath for 30 min before another centrifugation was performed to collect the resulting supernatant. A HPLC (LC-2030C, Shimadzu Corporation, Tokyo, Japan) was utilized for detection. The standard curves were created using a mixture of ATP, ADP, and AMP standards for comparison and quantification. The concentrations of ATP, ADP, and AMP were expressed in mg·kg^−1^ and the energy charge was calculated according to the formula:

$$Energy\;charge=\frac{ATP+ADP÷2}{ATP+ADP+AMP}$$

### 2.7. Determination of ATPase Activities

Assays for H^+^-ATPase, Ca^2+^-ATPase, and Mg^2+^-ATPase activities were conducted using 5g of randomly sampled *T. fuciformis* tissue, as per the methodologies detailed by Sun et al. and Li et al. [12,27]. First, 8 mL of extraction buffer solution (containing sucrose, ascorbic acid, glycerol, EDTA, DTT, and PMSF) was added to the *T. fuciformis* tissue and thoroughly mixed. After centrifugation, 0.2 mL of the supernatant was collected. Based on the determined ATPase activity, 0.5 mL of the corresponding reaction solution (H^+^-ATPase: MgSO_4_, Tris-HCl and KCl; Ca^2+^-ATPase: CaCl_2_, EDTA, DTT, Tris-HCl and NaCl; Mg^2+^-ATPase: MgCl_2_, EDTA, DTT, Tris-HCl and NaCl) was added and thoroughly mixed. Next, 0.1 mL of 1 mmol·L^−1^ ammonium molybdate solution and 0.2 mL of 5 mmol·L^−1^ ATP solution was introduced to the compound, which was then incubated in a 36 °C water bath for 20 min. Following that, 0.2 mL of 20% (*v*/*v*) trichloroacetic acid was immediately added after incubation, followed by centrifugation. Subsequently, 2 mL of ferrous sulfate-ammonium molybdate reagent was combined with the supernatant and vigorously shaken. The absorbance of the resulting ATPase enzyme extracts was measured at 660 nm. U·mg^−1^ protein is used to indicate the activities of the ATPases. 

### 2.8. Statistical Analyses

All experiments in this study were performed in triplicate, and the resulting data were imported into SPSS Statistics 22.0 software (IBM Corp., Armonk, NY, USA) for analysis. Differences between the treatment groups and control groups were evaluated using the T-test to assess the statistical significance. Data errors were represented by the STDEV.

## 3. Results

### 3.1. Changes in Disease Index

**Figure 1:**
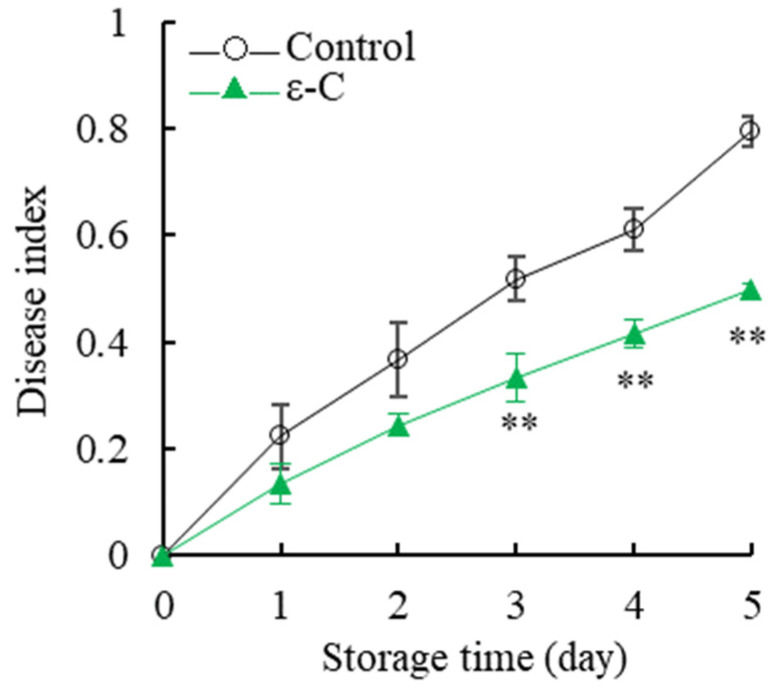
Changes in disease index of fresh *T. fuciformis* during the storage period. ** represents a level of significant difference of *p* < 0.01.

As illustrated in Figure 1, the postharvest disease index of fresh *T. fuciformis* demonstrated an increasing trend with the extension of storage time. The disease index of the control group increased sharply, reaching 0.79 on the fifth day of storage. In contrast, the treatment group’s disease index rose more rapidly during the early storage period (0–3 days) and then increased at a slower pace during the later period (3–5 days), consistently remaining lower than that of the control group. Further analysis revealed that the disease index of the treatment group was significantly lower than that of the control group on the second day of storage (*p* < 0.05) and remained significantly lower during the fourth and fifth days of storage (*p* < 0.01). These findings suggest that, compared to the control group, treatment with ε-C effectively delayed the increase of the disease index in postharvest fresh *T. fuciformis*, substantially reducing the occurrence of diseases during the storage period.

### 3.2. Changes in Weight Loss Rate

**Figure 2:**
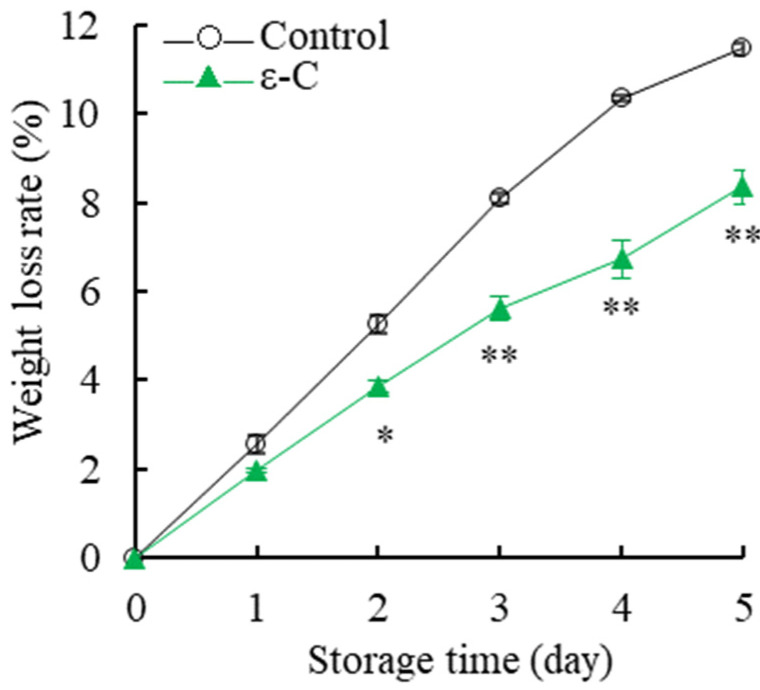
Changes in weight loss rate of fresh *T. fuciformis* during the storage period. * represents a level of significant difference of *p* < 0.05, ** represents a level of significant difference of *p* < 0.01.

The weight loss rate of postharvest *T. fuciformis* exhibited a continuous upward trend with increasing storage time, as depicted in Figure 2. During the five-day storage period, the control group’s weight loss rate accelerated rapidly, while the rate of increase in the treatment group was comparatively more gradual. Upon further comparison, it was found that, after five days of storage, the control group’s weight loss rate had increased by 11.48% compared to the day of harvest, while the treatment group’s weight loss rate only saw an 8.35% increase. Significance analysis indicated that the weight loss rate of the treatment group was significantly lower than that of the control group after two days of storage (*p* < 0.05) and remained very significantly lower during days 3–5 of the storage period (*p* < 0.01). The results demonstrated that treatment with ε-C effectively reduced the rate of weight loss in postharvest *T. fuciformis*, thereby effectively mitigating water loss and enhancing its shelf life.

### 3.3. Changes in Respiration Rate

**Figure 3:**
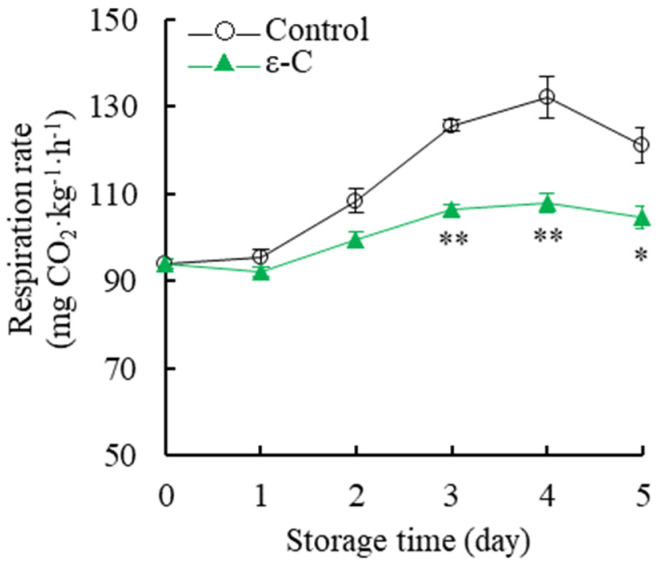
Changes in respiration rate of fresh *T. fuciformis* during the storage period. * represents a level of significant difference of *p* < 0.05, ** represents a level of significant difference of *p* < 0.01.

Figure 3 illustrates a trend in the postharvest respiration rate of fresh *T. fuciformis* that initially decreases, then increases, then is followed by a subsequent decrease. The respiration rate of the control group exhibited a rapid increase between the first and fourth days of storage, reaching a peak of 132.31 mg CO_2_ kg^−1^·h^−1^ on the fourth day of storage, while the treatment group recorded a lower peak at 107.78 mg CO_2_ kg^−1^·h^−1^. Statistical analysis indicated that the respiration rate of the treatment group was consistently significantly lower than that of the control group during days 3–4 of storage (*p* < 0.05), and significantly lower on the fifth day of storage (*p* < 0.01). The findings indicate that ε-C treatment contributes to the reduction of respiration intensity in postharvest fresh *T. fuciformis*, thus attenuating its physiological metabolic activity during storage and consequently extending its storage life.

### 3.4. Changes in Enzyme Activities of Respiratory Metabolism

**Figure 4:**
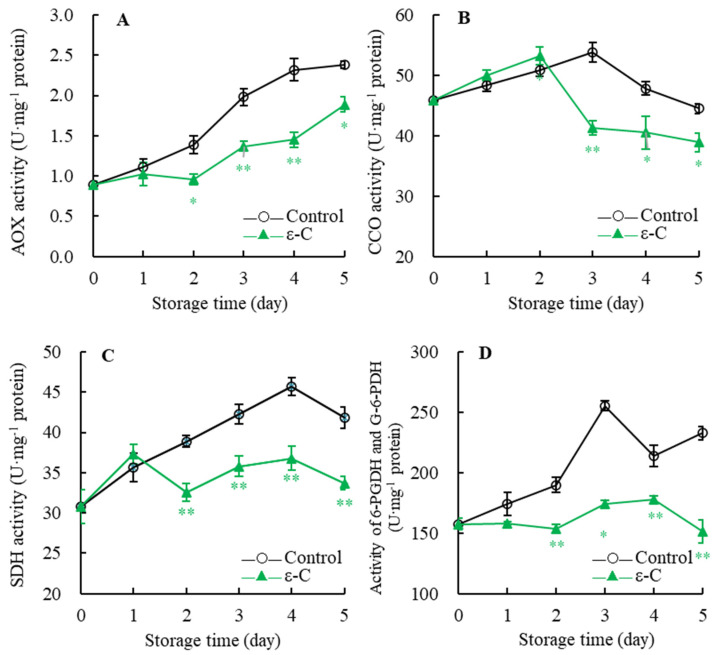
Changes in AOX (**A**), CCO (**B**), SDH (**C**), 6-PGDH and G-6-PDH (**D**) activities of fresh *T. fuciformis* during the storage period. * represents a level of significant difference of *p* < 0.05, ** represents a level of significant difference of *p* < 0.01.

As revealed in Figure 4A, the AOX activity in the control group showed a continuously rising trend with the extension of storage time. The AOX activity in the treated group increased slowly during days 0–1, declined slightly during days 1–2, and then surged during days 2–5. Throughout the entire storage period, the AOX activity in the treated group remained consistently lower than that in the control group, being 1.6 times higher in the control group on the fourth day of storage. Significance analysis demonstrated that the treated group’s AOX activity was significantly lower than the control group’s on the second and fifth days of storage (*p* < 0.05), and very significantly lower during days 3–4 of storage (*p* < 0.01).

Figure 4B indicates that CCO activity in the control group initially increased and then decreased during storage, peaking at 53.8 U·mg^−1^ protein on the third day. In contrast, the CCO activity in the treated group rose rapidly during days 0–2, dropped sharply during days 2–3, and then decreased slowly during days 3–5. The peak activity for the treated group occurred one day earlier than the control and remained lower than the control in the later stages of storage. Further analysis showed that the CCO activity in the treated group was significantly lower on the third day (*p* < 0.01) and it remained significantly lower during days 4–5 (*p* < 0.05) than it did in the control group.

From Figure 4C, it can be discerned that SDH activity in the control group rapidly increased during days 0–4, reaching the highest value of 45.7 U·mg^−1^ protein on the fourth day, and then swiftly declined. The SDH activity in the treated group surged during days 0–1, peaking at 37.3 U·mg^−1^ protein on the first day, followed by a steep decline during days 1–2, a gradual increase during days 2–4, and a rapid decrease during days 4–5. Statistical analysis revealed that the SDH activity in the treated group was consistently very significantly lower than that in the control group during days 2–5 of storage (*p* < 0.01).

As observed in Figure 4D, the activities of 6-PGDH and G-6-PDH in the control group increased modestly during days 0–2, surged during days 2–3, dramatically decreased during days 3–4, and then steeply increased during days 4–5. In the treated group, the activities of 6-PGDH and G-6-PDH slowly declined during days 0–2, slowly rose during days 2–4, and sharply decreased during days 4–5. Further comparison revealed that the activities of 6-PGDH and G-6-PDH were consistently higher in the control group during the entire storage period. Significance analysis showed that the activities of 6-PGDH and G-6-PDH in the treated group were very significantly lower than those in the control during days 2–5 of storage (*p* < 0.01). These results suggest that ε-C treatment can effectively slow the increase in AOX, CCO, and SDH activities, as well as 6-PGDH and G-6-PDH activities, in postharvest fresh *T. fuciformis*, maintaining lower enzymatic activities.

### 3.5. Changes in Energy Level

**Figure 5:**
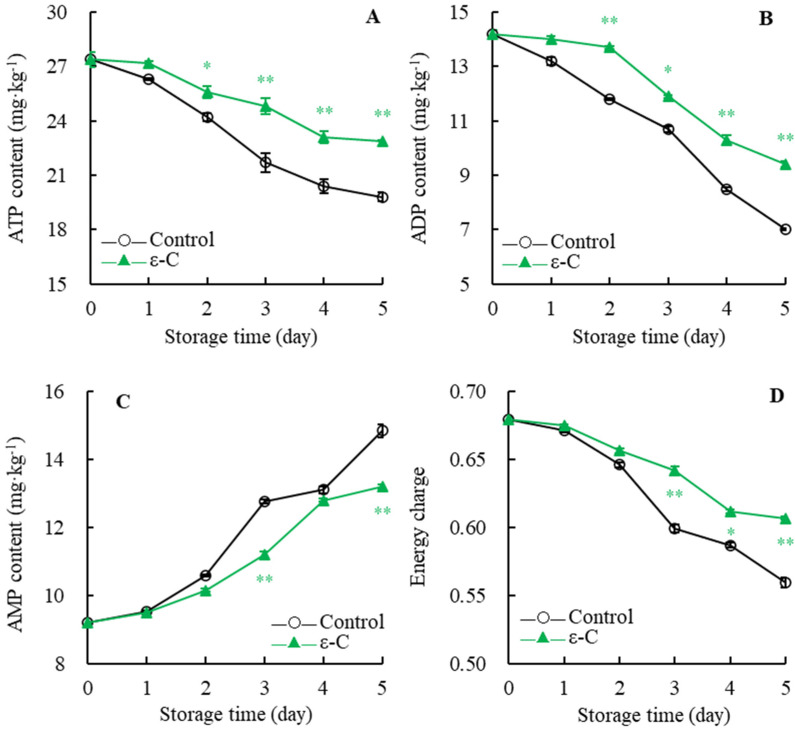
Changes in contents of ATP (**A**), ADP (**B**) and AMP (**C**), energy charge (**D**) of fresh *T. fuciformis* during the storage period. * represents a level of significant difference of *p* < 0.05, ** represents a level of significant difference of *p* < 0.01.

As depicted in Figure 5A, the ATP content trend in both the control and the treated groups during storage was essentially the same, showing a gradual decrease. Compared to the control group, the ATP content in the ε-C group was consistently higher throughout the storage period, with the treated group’s ATP content being 1.16 times that of the control group on day five of storage. Statistical analysis indicated that the ATP content in the treated group was significantly higher than that in the control group on day two of storage (*p* < 0.05), and highly significantly higher during days 3–5 (*p* < 0.01).

Figure 5B shows a continuous decline in the ADP content of the postharvest fruit in both the control and the treated groups. The rate of decline in the treated group’s ADP content was slow during days 0–2 and then accelerated during days 2–5. The control group exhibited a constant sharp decline, with the ADP content dropping to only 7 mg·kg^−1^ by day five. Further analysis revealed that the treated group’s ADP content was significantly higher than that of the control group on day three (*p* < 0.05), and very significantly higher on day two and during days 4–5 (*p* < 0.01).

As indicated in Figure 5C, AMP content of control group gradually increased during storage; it rose sharply during days 0–3, increased slowly during days 3–4, and then spiked again during days 4–5, resulting in an AMP content that was 1.61 times higher than it was on the postharvest day. In contrast, the AMP content in the treated group maintained a rapid increase during days 0–4 and then increased slowly during days 4–5. Significance analysis demonstrated that the treated group’s AMP content was very significantly lower than that of the control group on day three and day five (*p* < 0.01).

Figure 5D illustrates that the energy charge of both the control and the treated groups displayed a declining trend throughout the storage period. The energy charge in the treated group was consistently higher than in the control group, with the treated group’s energy charge decreasing to 0.607 on day five, while the control group’s fell to only 0.559. Statistical analysis showed that the energy charge in the treated group was significantly higher than that of the control group on day four (*p* < 0.05), and very significantly higher on day three and day five (*p* < 0.01). The results above indicate that, during the storage period, ε-C treatment of postharvest fresh *T. fuciformis* effectively inhibited the decrease in ATP and AMP contents, delayed the increase in AMP content, and maintained a higher level of energy charge.

### 3.6. Changes in ATPase Activities

**Figure 6:**
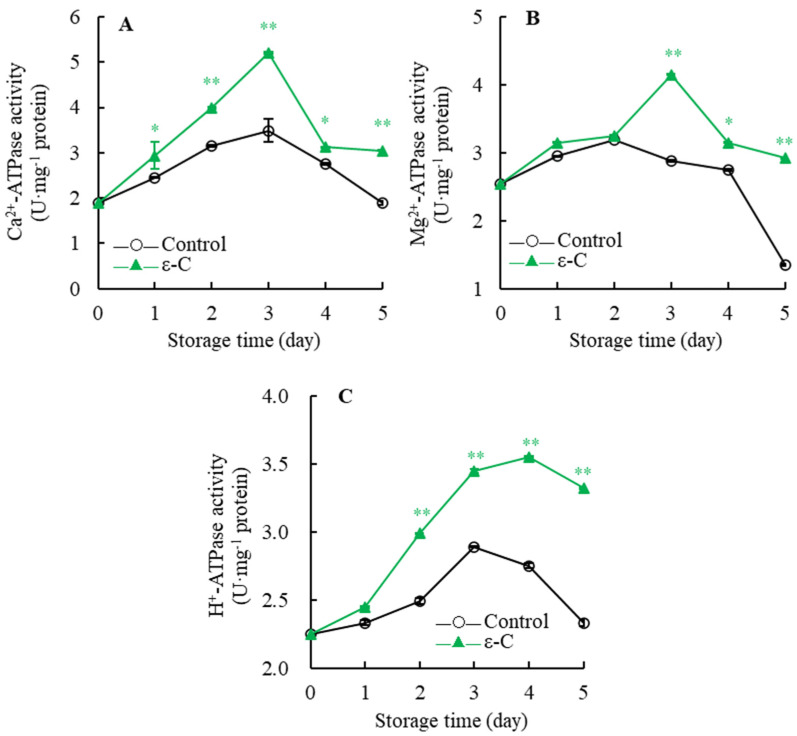
Changes in Ca^2+^-ATPase (**A**), Mg^2+^-ATPase (**B**) and H^+^-ATPase (**C**) activities of fresh *T. fuciformis* during the storage period. * represents a level of significant difference of *p* < 0.05, ** represents a level of significant difference of *p* < 0.01.

Figure 6A depicts that the Mg^2+^-ATPase activity in the control group incrementally increased during days 0–2 of storage, declined slowly during days 2–4, and then dramatically decreased during days 4–5. In contrast, the Mg^2+^-ATPase activity in the treated group gradually increased until day three, when it reached maximum activity; it then sharply declined during days 3–4, and slowly declined during days 4–5. Statistical analysis indicated that the Mg^2+^-ATPase activity in the treated group was significantly higher than that in the control group on day four of storage (*p* < 0.05), and very significantly higher on days three and five (*p* < 0.01).

As shown in Figure 6B, Ca^2+^-ATPase activity of control group slowly increased during days 0–3 and then rapidly decreased during days 3–5. Conversely, the Ca^2+^-ATPase activity in the treated group displayed a steep increase during days 0–3, then a sudden decline during days 3–4, and a gradual decrease during days 4–5. On day three, both groups’ Ca^2+^-ATPase activity reached their maximum values, with the treated group’s activity being 1.49 times that of the control group. Significance analysis revealed that the treated group’s Ca^2+^-ATPase activity was significantly higher than the control group on day one and day four (*p* < 0.05), and very significantly higher during days 2–3 and on day five (*p* < 0.01).

Figure 6C illustrates that the control group’s H^+^-ATPase activity exhibited a trend of initially increasing and then decreasing with the extension of storage time. The Mg^2+^-ATPase activity in the fruit flesh plasma membrane of the treated group increased dramatically during days 0–4, with the maximum value reaching 3.55 U·mg^−1^ protein on day four, which was a 1.58-fold increase from the postharvest day. Further analysis showed that the treated group’s Mg^2+^-ATPase activity was consistently very significantly higher than the control group’s during days 2–5 of storage (*p* < 0.01). These results suggest that ε-C treatment of postharvest fresh *T. fuciformis* can maintain higher activities of Mg^2+^-ATPase, Ca^2+^-ATPase, and H^+^-ATPase.

## 4. Discussion

After harvest, fruits, vegetables, and edible fungi must engage in respiration to obtain energy and maintain their metabolic balance. Intense respiratory activity, however, depletes energy and nutrients, subsequently leading to senescence and a decline in quality [28]. AOX and CCO are typical terminal oxidases in the respiratory chain and influence the level of respiration and the efficiency of electron transport within an organism [29,30]. In our study, following the ε-PL + chitosan coating (ε-C) treatment, the activities of AOX and CCO in *T. fuciformis* were lower than those in the control group, demonstrating a similar pattern to changes in disease index, weight loss, and respiration rate. These data substantiate that ε-C treatment can suppress the activity of terminal respiratory oxidases and reduce the rate of electron transfer and respiratory metabolism, thereby slowing down the consumption of energy and nutrients; and, consequently delaying the senescence and disease progression in *T. fuciformis*. Furthermore, the study of Li et al. showed that the application of high carbon dioxide and low oxygen treatment to *Pleurotus eryngii* slowed down the aging process, which may be attributed to the inhibited activity of terminal respiratory oxidases [23].

The changes in the activity of key respiratory metabolic enzymes can affect respiratory metabolic pathways such as the tricarboxylic acid (TCA) cycle and the pentose phosphate pathway (PPP), subsequently influencing the postharvest aging process [10]. SDH, a TCA cycle enzyme, catalyzes the cleavage of succinate’s carboxyl group while concurrently transferring two hydrogen atoms and two electrons to the cofactor flavin adenine dinucleotide (FAD); this reaction is an integral part of the TCA cycle, closely associated with oxidation and electron transfer processes [31]. 6-PGDH and G-6-PDH are two critical enzymes in the PPP. G-6-PDH catalyzes the transformation of glucose-6-phosphate into 6-phosphogluconolactone while generating the reduced form of nicotinamide adenine dinucleotide phosphate (NADPH), and 6-PGDH converts 6-phosphogluconolactone into ribulose 5-phosphate, sustaining the function of the PPP [31,32]. In our study, the SDH, 6-PGDH and G-6-PDH activities of *T. fuciformis* treated with ε-C were lower than those of the control group. We hypothesize that ε-C treatment suppresses respiratory metabolism by reducing the proportion of the TCA cycle and the portion of the PPP, ultimately delaying the senescence of *T. fuciformis*. Moreover, reports by Li et al. have also indicated that aging progression in straw mushroom (*Volvariella volvacea*) can be suppressed by reducing the activities of 6-PGDH and G-6-PDH, thereby decreasing the proportion of the PPP pathway [25].

The energy state of postharvest fruits, vegetables, and edible fungi has a profound impact on their preservation. Once harvested, they transition from a state of growth to one of senescence and decomposition, commencing the continuous consumption of energy substrates [26]. A higher energy state indicates the presence of sufficient substrates within the cells to maintain a relatively slow respiration rate, which can effectively delay the depletion of energy and tissue senescence. Within this context, ATP provides the energy required for various physiological processes, including active transport, macromolecule synthesis, cell division, and various metabolic pathways. Moreover, the levels of ADP, AMP, and the energy charge are critical markers for evaluating the energy state [12,33,34]. In this experiment, *T. fuciformis* treated with ε-C had higher contents of ATP and ADP and an elevated energy charge, as well as lower levels of AMP and a lower disease index and weight loss rate, compared to the control group. These results suggest that ε-C treatment can slow down the consumption of energy, maintain the integrity of the cellular structure of *T. fuciformis*, and delay its senescence and disease progression. Similarly, Yao et al. have proposed that Nanocomposite-based packaging treatment can slow down the decline in ATP content and energy charge in white *Hypsizygus marmoreus*, thereby inhibiting the quality loss of white *H. marmoreus* [35].

Ca^2+^-ATPase, Mg^2+^-ATPase, and H^+^-ATPase are crucial enzymes in the process of energy metabolism, playing roles in the hydrolysis of energy from ATP molecules to facilitate the transport of various molecules across cell membranes, intracellular signal transduction, and other biological processes [36]. Moreover, these ATPases are significant for the formation and maintenance of the electrochemical gradient across cell membranes, as well as for sustaining cellular pH and osmotic pressure. Thus, maintaining high ATPase activity is essential for the preservation of intracellular energy [37]. In this study, the activities of Ca^2+^-ATPase, Mg^2+^-ATPase, and H^+^-ATPase in *T. fuciformis* treated with ε-C were observed to be higher than those in the control group. When coupled with previous experimental results, these data indicate that novel water-based phase change coolant (PCC) treatment can enhance ATPase activities and maintain an optimal level of energy supply, thereby delaying the senescence of shiitake mushrooms (*Lentinula edodes*) [38].

Moreover, it has been found that the application of a chitosan-based composite coating effectively prolongs the shelf life of and combats bacterial contamination in shiitake mushrooms (*Lentinus edodes*) and enoki mushrooms (*Flammulina velutipes*) [18,39]. These findings indicate that composite coatings possess considerable research merit and application potential within the realm of edible fungi preservation.

## 5. Conclusions

Studies indicate that ε-PL and chitosan composite coating preservatives can effectively delay senescence and quality deterioration of fresh *T. fuciformis* during storage. Further analysis reveals that these preservatives can suppress the electron transport chain and the TCA cycle and PPP in the respiratory metabolism, thereby moderating the respiration rate through the alteration of respiratory metabolic pathways. Additionally, the treatments are observed to enhance the activities of Ca^2+^-ATPase, Mg^2+^-ATPase, and H^+^-ATPase, thus elevating the energy levels of *T. fuciformis* during storage. Consequently, it is believed that ε-PL and chitosan composite coatings hold significant potential for the preservation of fresh edible fungi.

## Figures and Tables

**Table 1:** The evaluation method of disease index.

Level	Characteristics
0	disease-free
1	less than 25% of the *T. fuciformis* lobes have few disease spots
2	less than 50% of the *T. fuciformis* lobes have few disease spots or less than 25% of the lobes have several disease spots
3	less than 75% of the *T. fuciformis* lobes are diseased, or less than 25% of the lobes are completely rotted
4	over 75% of the *T. fuciformis* lobes are diseased, less than 50% of the lobes are completely rotten, or the entire *T. fuciformis* is rotten

## Data Availability

The original contributions presented in the study are included in the article, further inquiries can be directed to the corresponding author.
